# *Enterococci* Mediate the Oviposition Preference of *Drosophila melanogaster* through Sucrose Catabolism

**DOI:** 10.1038/s41598-017-13705-5

**Published:** 2017-10-18

**Authors:** Wei Liu, Ke Zhang, Yujuan Li, Wanzhen Su, Kunkun Hu, Shan Jin

**Affiliations:** 1 Department of Medical Laboratory Science, Fenyang College, Shanxi Medical University, Shanxi, 032200 China; 2 Department of Clinical Medical, Fenyang College, Shanxi Medical University, Shanxi, 032200 China; 3 Department of Cell Biology, College of Life Science, Hubei University, Hubei, 430062 China

## Abstract

Sucrose, one of the main products of photosynthesis in plants, functions as a universal biomarker for nutritional content and maturity of different fruits across diverse ecological niches. *Drosophila melanogaster* congregates to lay eggs in rotting fruits, yet the factors that influence these decisions remains uncovered. Here, we report that lactic acid bacteria *Enterococci* are critical modulators to attract *Drosophila* to lay eggs on decaying food. *Drosophila*-associated *Enterococci* predominantly catabolize sucrose for growing their population in fly food, and thus generate a unique ecological niche with depleted sucrose, but enriched bacteria. Female flies navigate these favorable oviposition sites by probing the sucrose cue with their gustatory sensory neurons. Acquirement of indigenous microbiota facilitated the development and systemic growth of *Drosophila*, thereby benefiting the survival and fitness of their offspring. Thus, our finding highlights the pivotal roles of commensal bacteria in influencing host behavior, opening the door to a better understanding of the ecological relationships between the microbial and metazoan worlds.

## Introduction

In nature, animals must navigate a complicated and ever-changing environment for survival and reproduction^1^. Owing to the vulnerability and the restricted mobility of larvae, selecting appropriate egg laying sites is especially significant for survival and fitness of offspring in generalist insects, including *Drosophila melanogaster* ^2,3^. Wild *Drosophila* is notorious for being attracted to depositing their eggs into decaying fruits, thereby providing an attractive model to study the oviposition selection. Interestingly, most *Drosophila* resists unripe fruits, due to low nutrition and high toxic secondary metabolites^4^. However, fruits undergo a ripening process that converts the firm tissue into soft, sugar-rich ones and neutralizes toxin, eventually contributing to the high nutritional value of mature fruit^5^. Ripe fruits are susceptible to a myriad of fermenting bacteria, and the amount of bacteria increase their population by consuming sugars in rotting fruit. Roiled ripe fruit become decomposed, but are attractive to saprophytic animals, like *Drosophila*. Emerging studies have sparked the notion that microbiota have profound impacts on neurodevelopment, the central nervous system, and behaviors^6^. Despite a wealth of knowledge about the fly-microbe relationship, little is known about how *Drosophila* behavior is influenced by the rotting fruit-associated microbiota. The powerful genetic tools in *Drosophila*, coupled to low microbiota complexity, makes *Drosophila* an ideal host model to tackle this challenging problem^7^.

Lactic acid bacteria (LAB) are a clade of gram-positive, acidophilic bacteria that are widespread on nutrient-rich resources and animal hosts, including humans. They are also prevalent commensals of *Drosophila*, which are mainly represented by the genera *Lactobacillus* and *Enterococcus* ^8^. LAB share common metabolic characteristics that consume hexoses to produce lactate as the major metabolic end product of fermentation. Food fermentation processes change food tastes as well as volatile odors, which are both used to assess the nutritional quality of food by animals. Studies have shown that *Enterococci* affect a wide spectrum of host physiological traits^9–12^, but how it influences fitness-related behaviors is poorly understood. Food odors help the animal to track down food over long distances, while tastes are ultimately crucial to making decisions to feed on or to lay an egg over short distances. Most studies have exploited the fact that food odors trigger the oviposition preference of females^13–15^, but roles of tastants in influencing oviposition behavior are almost ignored. Given that tastants are also molecular cues that are translated into appropriate behaviors via the gustatory system^16^, it is assumed that *Drosophila*-associated LAB could affect their host behaviors by altering food tastes. Using *E*. *faecium* and *Drosophila* model, we developed a fly food fermentation system that afforded the measurement of microbial metabolism and host ovipositional behavior.

With a surge of interest that microbiome shapes behavior across many animal taxa, we attempted to investigate the roles of LAB in the oviposition preference of *Drosophila* under the laboratory and natural conditions. Herein, we report that commensal *E*. *faecium* allures *Drosophila* to lay eggs on fermented food. *E*. *faecium* predominantly consume sucrose in food, and consequently generate new sites with lower sucrose that acts as an oviposition guidance cue for females, assisting *Drosophila* in finding a rich source of bacteria. Our results revealed that commensal bacteria could be an integral contributor to the oviposition preference of *Drosophila*, providing an insight into the ecological and evolutionary dynamics that shape these communities.

## Results

### Egg laying preference for fermented food

**Figure 1:**
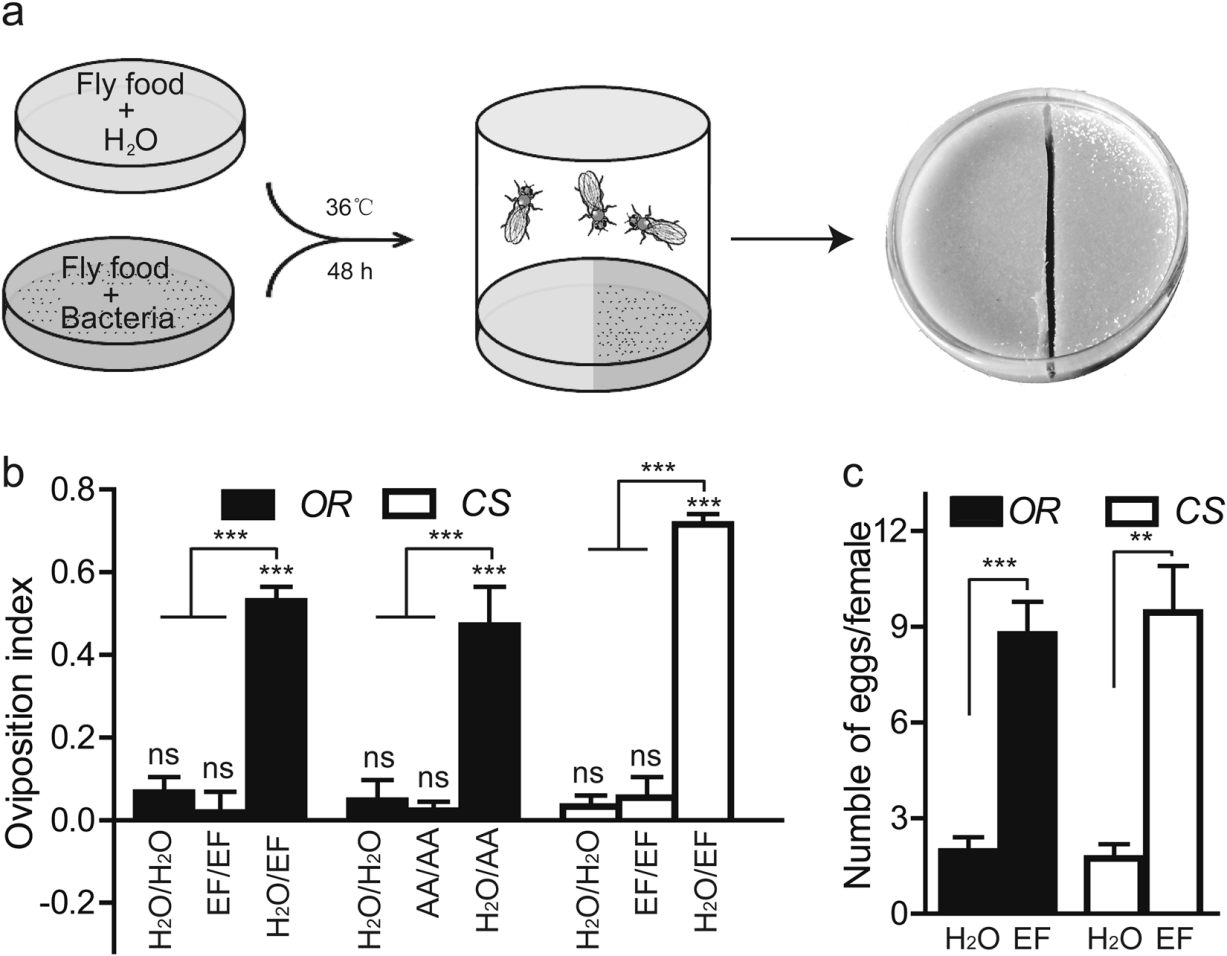
The innate oviposition behavior in response to fermented diet. (**a**) A diagram of the egg laying preference assay with the 2-choice cage. The surface of fly food was augmented with bacteria to generate a fermented diet for 48 h in the incubator, whereas the control was used with H_2_O. Each food item was chopped into two halves, and each half was placed into the 2-choice cages. Mated females with yeast paste were transferred to the 2-choice cage and allowed to lay eggs for 16 h. The numbers of eggs were counted on each half, and the oviposition preference was calculated. (**b**) The quantification of egg laying preference for fermented fly food by wild-type Oregon R (OR) and Canston S (CS). H_2_O: water, EF: *Enterococcus faecium*, AA: acetic acid; mocks are two halves of fly food with water or EF). The one-sample *t*-test was used to assess the mean deviance of each column from 0; ANOVA tests with LSD post hoc analysis were used to calculate significant differences between columns, n = 6–14. (**c**) The stimulation of egg laying with fermentation. Twenty females were transferred into each cage of the whole-forced cage with a control or fermented diet, respectively, and the average number of eggs was calculated. ANOVA tests with LSD post hoc analysis, n = 6–8. Mean ± SEM; Symbols: NS p > 0.05; *p < 0.05; **p < 0.01; ***p < 0.001.

In the environment, adult females frequently lay eggs on rotting fruits comprised with various microbes, proposing that microbes could attract flies to lay eggs. To this end, we developed a system of fly food fermentation with bacteria (Fig. 1a), and the oviposition preference of *Drosophila* females was evaluated in 2-choice cages as described^17^. Of note, protein source yeast was replaced with casamino acids (casein) in the fly food recipe, precluding any side effect of microbial metabolites on host behaviors^18^. Fascinatingly, our data shown that wild-type Oregon R females laid approximately 76% of their eggs on fermented halves, and the oviposition index (OI) was 0.53 (Fig. 1b). No bias of oviposition preference was observed in two mocks in the apparatus (Fig. 1b), but the ovipositional attractiveness to acetate, the positive control, agreed with one study^17^. This result indicated that females were prone to select fermented food for their egg laying, congruent with ecological phenomena of oviposition behavior. Consistently, wild-type Canton S females also displayed the oviposition preference for fermentation with OI of 0.72 (Fig. 1b), suggesting that this oviposition preference didn’t arise from genetic variation. Analogously, the bias of egg laying to fermentation recurred in the larger arena that allowed 300 female flies more freedom of movement (Supplementary Figure 1a,b). Interestingly, flies given no choice but to lay eggs on the whole-forced cage laid 4.5-fold more eggs on fermented food compared to control food (Fig. 1c), suggesting that the oviposition preferences observed in the two-choice assay came directly from the capacity of fermentation to elicit egg laying. The ovipositional allurement was also observed in Canton S fly (Fig. 1c). Because two strains of *Drosophila* responded similarly, a representative Oregon R was used to test the effect of *E*. *faecium* on *Drosophila* behavior. Taken together, our results demonstrated that the presence of indigenous *Enterococci* induced the *Drosophila* oviposition preference.

### The preference for fermentation is specific to oviposition

**Figure 2:**
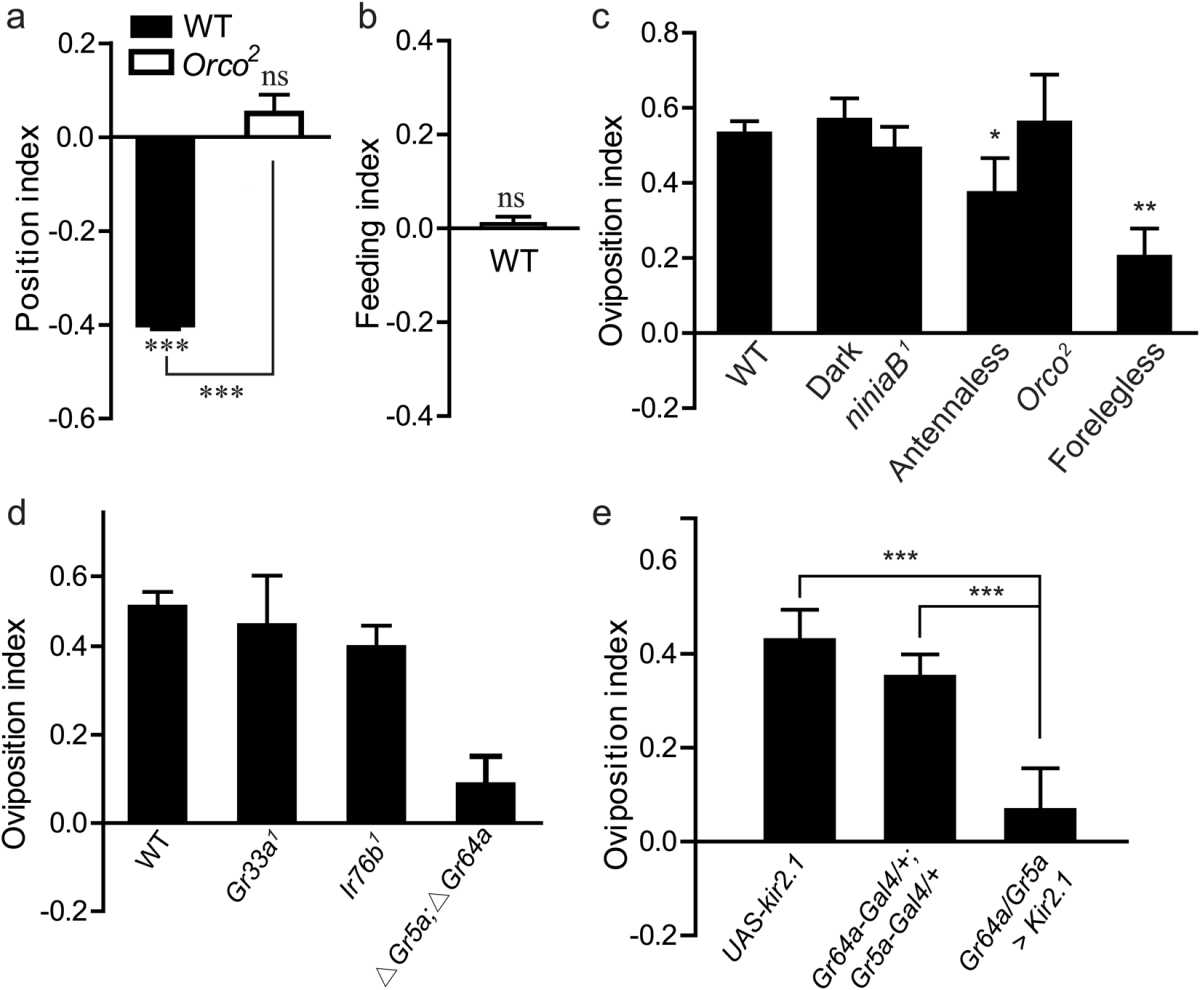
Role of gustatory system and sucrose receptor neurons in oviposition choices. (**a**) The positional preference for fermentation. Wild-type (WT)  flies were averse to a fermented fly food, while *Orco* ^2^ mutants deficient in odor were neutral. Females were presented the following 2-choice as depicted in Fig. 1a, and position was examined. The one-sample *t*-test was used to assess the mean deviance of each column from 0; ANOVA tests with LSD post hoc analysis were used to calculate significance differences between columns; n = 28–46. (**b**) The feeding preference for fermented food. Females were presented the following 2-choice food combinations, and one half food was supplemented with dye. After feeding, the amount of dye in fly gut contents was quantified. The one-sample *t*-test was used to assess the mean deviance of each column from 0; n = 8. (**c**) Screening of candidate sensory modalities for oviposition selection for fermentation. The indicated animals were allowed to choose using two-way food preference assays. For vision, WT flies in darkness and *ninaB* ^1^ were used; for olfaction, antennaectomized females (surgically removing the primary olfactory organs) and *Orco* ^2^ mutants (unable to respond to most olfactory stimuli) were used; for gustation, the forelegs that contain gustatory sensilla were surgically ablated. ANOVA tests with LSD post hoc analysis, n = 6–12. (**d**) The role of *Gr5a; Gr64a* neurons in the oviposition preference for fermentation. *Gr33a* ^1^ mutants, *IR76b* ^1^ mutants and Δ*Gr5a;* Δ*Gr64a* double mutants were used, and the oviposition index was evaluated. ANOVA tests with LSD post hoc analysis, n = 8–15. (**e**) Hyperpolarizing *Gr5a* and *Gr64a* neurons reduced the oviposition preference for fermentation. Animals carrying *Gr64a-GAL4;Gr5a-GAL* or *UAS-Kir2*.*1* were used as a negative control. Mann-Whitney Test, n = 5–8. Mean ± SEM. Symbols: NS p > 0.05; *p < 0.05; **p < 0.01; ***p < 0.001.

Based on the fact that females continue to seek the suitable sites to deposit eggs after one egg laying event^19^, it was postulated that oviposition preference would stem from the positioning and feeding attractiveness to fermentation. To record the physical location of flies, we conducted a positioning assay in 2-choice cages depicted in Fig. 1a ^17^. Unanticipatedly, females were strongly repelled to the fermented food, with a positional index of −0.40 (Fig. 2a). The opposition of ovipositional and positional drivers ruled out the possibility that the oviposition preference for fermentation was attributed to the positional attraction. Next, we investigated the feeding preference for fermentation using a modified 2-choice assay. Flies ingested approximately equal amounts of food in two halves (Fig. 2b), suggesting that feeding preferences did not elicit oviposition-site selection for fermentation. Thus, these results suggested that the oviposition preference for fermentation was *bona fide* derived from oviposition-site selection.

### The gustatory system mediates the ovipositional attraction to fermentation

**Figure 3:**
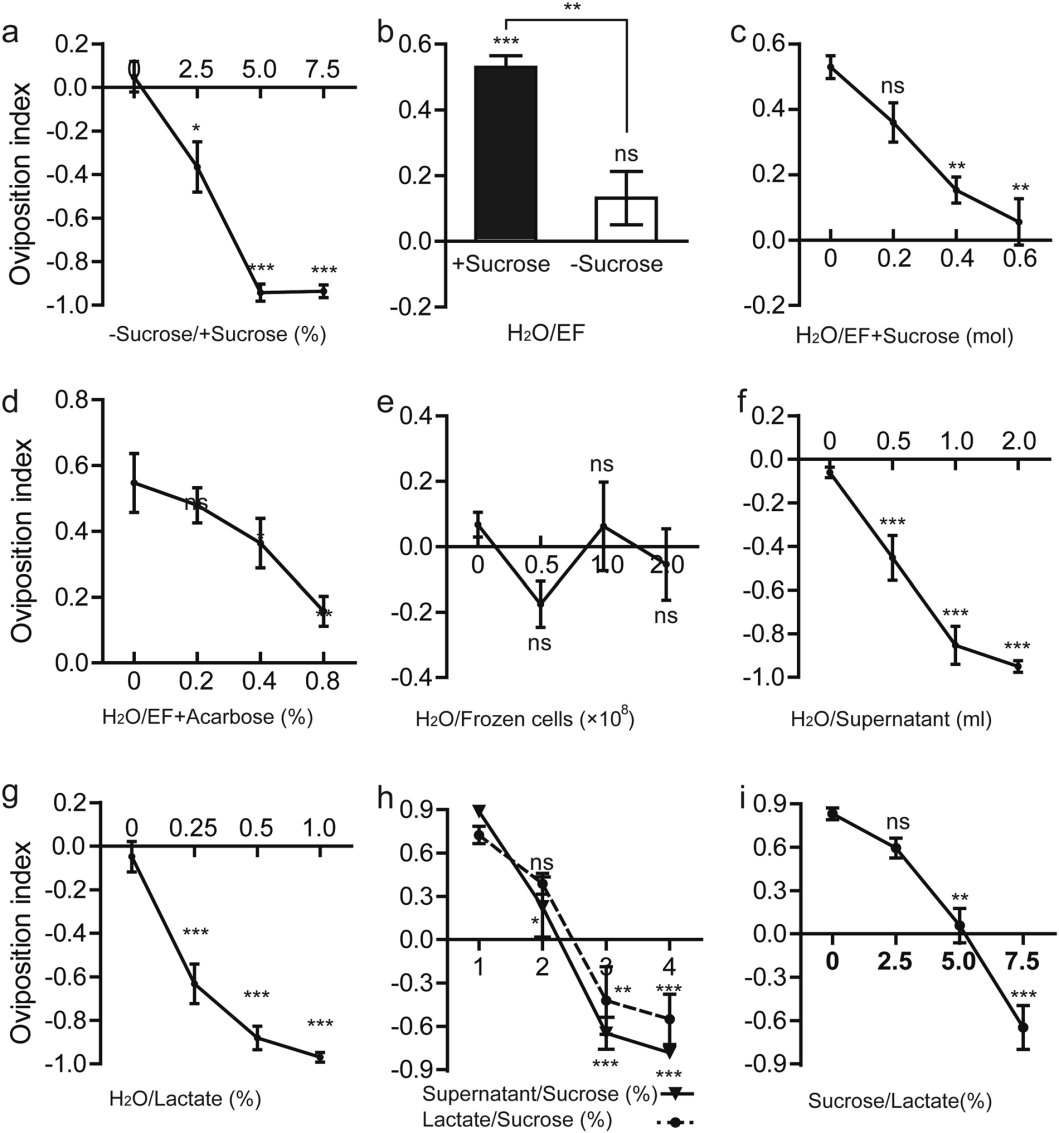
Sucrose modulated the oviposition preference for fermentation. (**a**) Females avoided to deposit eggs on the casein-cornmeal-agar media titrated to different sucrose (+Sucrose%) compared to fly food without sucrose (−Sucrose). n = 12, one of two replicates. (**b**) Sucrose deprivation impaired the oviposition preference for fermentation. Fly food was control food (+sucrose) or deprived of sucrose (−sucrose). The 2-choice cage of each food was assembled with H_2_O or bacteria. n = 8, one of three replicates. (**c**) Sucrose replenishment attenuated this oviposition preference in a dose-dependent manner. Sucrose was added to fermented fly food (EF + Sucrose), and the oviposition preference of EF + sucrose food was compared to fermented food (H_2_O). ANOVA tests with LSD post hoc analysis were used to calculate significant differences between columns. n = 12, one of two replicates. (**d**) The α-glucosidase inhibitor, acarbose, diminished the oviposition preference for fermentation. Acarbose was added to fermented fly food (EF + acarbose), and the oviposition preference of EF + acarbose food was compared to fermented food (H_2_O). n = 12, one of two replicates. (**e**) Bacterial cells were dispensable to trigger the oviposition preference for fermentation. Frozen bacterial cells were supplemented on the surface of one half of fly diet in a 2-choice cage, and ovipositional preference for bacterial cells was compared to fly food with water. n = 12, one of three replicates. (**f**,**g**) Fruit flies were averse to laying eggs on the media with LAB metabolites or lactate. n = 12, one of three replicates. Supernatant or lactate was added to one half of fly diet in a 2-choice cage, and the ovipositional preference for them was compared to fly food with water (H_2_O), respectively. (**h**) Sucrose was a more robust factor that suppressed the oviposition of females than EF metabolites or lactate. Females were allowed to choose between 0.5 ml LAB metabolites (Supernatant) or 1% lactate (Lactate) and dosage-dependent sucrose (Sucrose%) using 2-choice food preference assays. High concentration sucrose reversed the avoidance to LAB metabolites and sucrose. n = 12, one of three replicates. (**i**) The aversion to 5% sucrose (Sucrose) was affected by lactate in a dosage-dependent manner (Lactate%). Significance was calculated by ANOVA tests with LSD post hoc analysis for Fig. 3e and ANOVA tests with LSD post hoc analysis for others. Mean ± SEM. Symbols: NS p > 0.05; *p < 0.05; **p < 0.01; ***p < 0.001.

Sensory modalities of gustation, vision and olfaction are orchestrated to translate environmental cues into appropriate behaviors. To uncover the neural basis of the fly’s attraction to fermentation, we sought to screen the sensory modality for fermentation sensation. We firstly assessed the roles of vision in ovipositional preference with *ninaB* mutants with the elimination of vision. The result showed that *ninaB* retained an oviposition preference for fermenting food with an OI of 0.49 (Fig. 2c). Likewise, females in the dark boxes retained an oviposition preference for fermented food (Fig. 2c). The results suggested that the general vision receptor was not required for fermentation sensing. Because volatile chemicals are known modulators of many social behaviors, we next analyzed the behavior of putative anosmic flies. *Orco* ^2^ mutants, unable to respond to most olfactory stimuli, didn’t alter the egg laying preference for fermented media (Fig. 3c), indicating that the olfactory system was dispensable for the ovipositional behavior. Flies with surgically removed antennae also displayed the evident preference of oviposition, albeit of the lower preference compared to the intact fly. Taken together, neither vision nor olfaction accounted for this behavior (Fig. 2c).

Fly forelegs contain gustatory receptors and function as one of the primary gustatory organs^20^. In order to evaluate roles of the gustatory system in oviposition, we surgically removed forelegs to partially impair their gustatory system. The bias to fermentation of forelegless females was dramatically decreased (Fig. 2c), implying that the gustatory system was presumably responsible for the oviposition preference for fermentation. Taste sensilla contain several types of gustatory receptor neurons (GRNs), including bitter-, salt-, and sugar-sensitive neurons. To identify specific GRNs, we carried out a small genetic screen using loss of function of single GRNs. We found that either *Gr33a* ^1^ mutants (bitter) or *IR76b* ^1^ mutants (salt) were normally attracted to lay eggs in fermented halves of diet (Fig. 2d). The results suggested that neither bitter sensing nor salt sensing accounted for the oviposition preference for fermentation. In contrast, Δ*Gr5a;* Δ*Gr64a* double mutants completely lost their attraction to oviposit in the fermented diet (Fig. 2d). The sweet substance receptor (*Gr5a* and *Gr64a*) neurons conduct sucrose sensing^2,21,22^, indicating that *Gr5a* and *Gr64a* neurons specifically accounted for the oviposition preference for fermentation. To strengthen the evidence, we inhibited synaptic transmission by expressing a hyperpolarizing *Kir2*.*1* potassium channel in *Gr5a* and *Gr64a* sensory neurons. *Gr5a-GAL4/UAS-Kir2*.*1*; *Gr64a-GAL4/*+ flies displayed an impaired oviposition preference torward fermentation, whereas parent fly controls apparently exhibited the preference (Fig. 2e). We, hence, concluded that sucrose sensing through the *Gr5a* and *Gr64a* neurons mediated oviposition behavior for fermentation in *Drosophila*.

### Sucrose mediating oviposition preference for fermentation

Sucrose is rich in ripe fruits, but almost depleted by fermenting microbes in rotting fruit, providing a common biomarker for nutrient content and fermentation status across diverse ecological niches. Under laboratory conditions, fly food usually contains 5–10% sucrose (w/v) that is supposed to offer flies energy. Earlier findings showed that sucrose acts as a negatively potent cue that guides the selection of oviposition site^2^. In a binary oviposition choice assay, it was ascertained that sucrose was sufficient to suppress females to lay eggs on the casein-cornmeal-agar diet (Fig. 3a). This result conflicts with the universal rule that animals prefer calorie-dense food, and remains unresolved. In fact, sucrose was efficiently converted to lactate by LAB during fermentation (Supplementary Figure 2), prompting us to test whether sucrose turnover could potentially underlie the finding that *Drosophila* was attracted to lay eggs in fermented food. For this purpose, we assessed the effects of the absence of sucrose on the oviposition preference during the fermentation process. Indeed, sucrose deprivation led to an indistinguishable oviposition preference for fermented food compared to control in absence of sucrose (OI = 0.13, Fig. 3b), indicating that sucrose played a critical role in the oviposition preference for fermentation. To further verify it, we supplemented the fermented halves with doses of sucrose, making the final concentration of sucrose on fermented medium comparable to control. This oviposition preference was substantially abolished by the addition of sucrose (Fig. 3c). Finally, we set up the oviposition preference tests with acarbose, an inhibitor of alpha-glucosidase, that decreases sucrose hydrolysis. Our data showed that acarbose attenuated the oviposition preference of females in a dosage-dependent manner (Fig. 3d). Together, our data demonstrated that sucrose was a key factor that contributed to the oviposition preference toward fermentation. Interestingly, we found that flies had no preference for bacterial corpses (Fig. 3e), and were repelled to deposit their eggs on halves with a supernatant of casein-cornmeal-sugar liquid medium fermented by *E*. *faecium* (Fig. 3f). These findings argue that neither bacterial cells nor their anabolic products were required for oviposition choice for fermentation. Instead, we hypothesized that metabolic products of fermentation were the key determinants of oviposition choice. Given that lactate is the major metabolic end product of LAB fermentation, we tested and confirmed that lactate recapitulated the oviposition aversion of females to the supernatants of fermented fly food (Fig. 3g). This data further supports that bacterial anabolites didn’t contribute to this oviposition preference.

### Sucrose is a robust factor for ovipositional selection

Since females avoided sucrose, supernatant and lactate, we further compared the relative repellency of them against ovipositional selection in the 2-choice assays. As shown in Fig. 3h, the 2-choice assay was carried with diets with supernatant of fermented food or 1% lactate and sucrose in a dosage-dependent manner. Our data showed that 4% or more than sucrose efficiently overwhelmed the oviposition aversion to either LAB supernatant or lactate. The shifts suggested that sucrose, compared with the metabolites of LAB or lactate, was a more robust factor that influenced the oviposition preference of *Drosophila*. In a reverse assay (Fig. 3i), the oviposition aversion to sucrose endured a high concentration of lactate that was less than 2% in naturally fermented media. In sum, our data demonstrated that sucrose was a robust regulator of oviposition preference, and the depletion of sucrose by commensal bacteria efficiently generated the oviposition preference for fermentation.

### The relationship of sucrose consumption and bacterial population with oviposition preference

**Figure 4:**
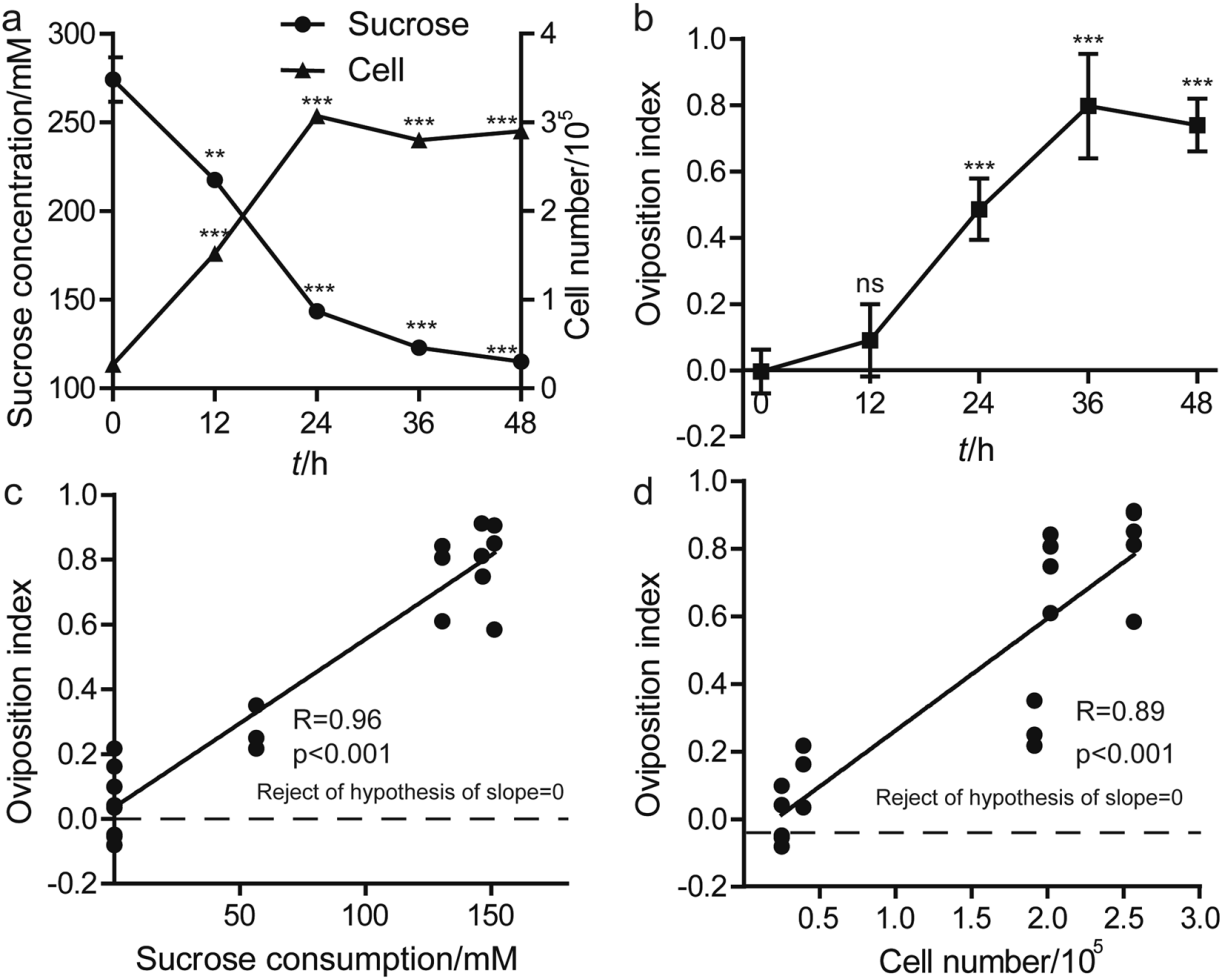
*Drosophila* preference for fermentation correlated with sucrose consumption and bacterial population. (**a**) The concentration of sucrose and viable bacterial cells in fly food. The concentrations of sucrose reduced over time, while bacterial density increased. n = 12, one of three replicates. (**b**) *Drosophila* temporal oviposition preference for fermentation. n = 16, one of three replicates. (**c**,**d**) The relationship between sucrose consumption, bacterial density and *Drosophila* oviposition preference for fermentation. A linear standard curve with an unconstrained slope was generated and compared to a null model with slope = 0. Each data point represents sucrose concentration or viable cell number of fly food along with the mean oviposition index value toward fermentation. A semilog standard curve with an unconstrained slope was generated and compared to a null model with slope = 0. The data fit to an unconstrained slope better than to the null model (For sucrose consumption: p < 0.0001, slope = −0.95; for bacterial density: p < 0.0001, slope = −0.95). ANOVA tests with LSD post hoc analysis. Mean ± SEM. Symbols: NS p > 0.05; **p < 0.01; ***p < 0.001.

Since bacteria dominate fly food and metabolize sucrose during fermentation, we further examined sucrose content and bacteria population in the media over time. Expectedly, the sucrose concentration of fly media decreased, while the density of bacteria increased (Fig. 4a), indicating that fermenting bacteria increased their population through sucrose catabolism. We next evaluated the intrinsic capacity of each stage to elicit oviposition. In agreement with this, *Drosophila* oviposition preference toward fermentation time rose, and eventually remained stable up to 36 h (Fig. 4b). To appreciate the relationship of sucrose consumption (real-timing sucrose concentration – original sucrose concentration) and oviposition preference toward fermentation, we calculated them with a regression line in a Graphpad software. The unconstrained slope of a linear standard curve was 0.96 compared to 0 in a null model, indicating that the *Drosophila* oviposition preference index was positively correlated with sucrose consumption (Fig. 4c). In the meantime, *Drosophila* oviposition preference index was correlated with bacterial population density (Fig. 4d). Notably, it was likely that flies found these egg laying sites through the lower sucrose rather than bacterial population, because flies were not attracted to bacterial cells (Fig. 3e). These results suggest that *Drosophila* probed lower sucrose cues to seek suitable oviposition sites with higher bacterial populations. Hence, the metabolism of sucrose by commensal bacteria could help alert flies to the presence of abundant bacteria cells.

### Sucrose-fermenting bacterial community in rotten fruits

**Figure 5:**
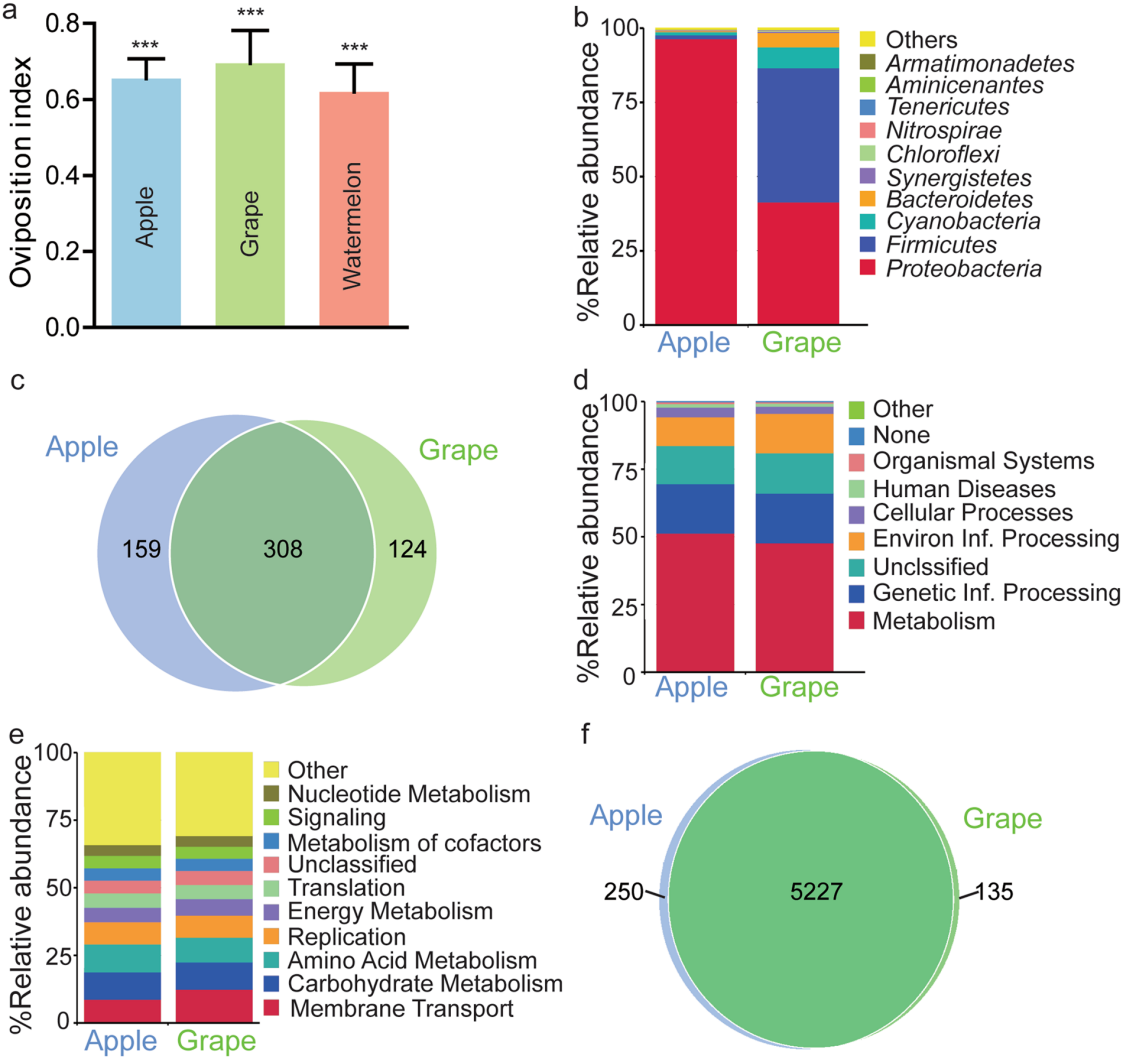
The composition and function of rotting fruit-associated microbiota. (**a**) Two-choice oviposition assay for fruit purée. Fruit purée (20%w/v) replaced sucrose in the behavior-testing media, and oviposition preference was assayed between fresh and fermented choices. The one-sample *t*-test was used to assess the mean deviance of each column from 0. n = 4. (**b**) Composition and distribution of the dominant bacterial taxa within rotting apples and grapes. (**c**) Venn diagram showing the presence of bacterial taxa within two fruits. The number of bacteria in rotting apples and grapes was in the circles. (**d**,**e**) PICRUSt predicted microbiota function based on inferred metagenomes of rotting fruit-associated bacteria at the primary (**d**) and upper (**e**) level using the PICRUSt algorithm. (**f**) Venn diagram showing the distribution of predicted KEGG genes within two fruit samples. The one-sample *t*-test, Mean ± SEM, Symbols: ***p < 0.001.

Ripe fruits, like apples and grapes, are a rich source of sucrose and are more susceptible to microbial decay. To examine its influence on oviposition site selection, we used either apple or grape purée to replace sucrose in behavior testing media. This oviposition bias to fermentation persisted on media with apple, grape or watermelon purée (Fig. 5a), prompting us to decipher the diversity of bacterial community and metabolic profile in rotten fruits. Rotting apples and grapes were collected from orchards during the fruit harvest season, and were subjected to deep sequencing of the 16 S rRNA genes. With the average neighbor algorithm with 97% sequence similarity, clustering created 513 and 448 operational taxonomic units in apple and grape samples (Supplementary Dataset), respectively. In apple and grape samples, the fermenting bacteria were diverse and dominated by bacteria of two phyla: Proteobacteria and Firmicutes (Fig. 5b). The most dominant two bacteria were *Gluconobacter* (80%) and *Acetobacter* (6%) in apple samples, and *Fructobacillus* (38%) and *Acetobacter* (15%) in grape samples (Supplementary Dataset). Indeed, most dominant bacteria, belonging to LAB and acetic acid bacteria, are well-known fermentative bacteria that convert many sugars to acids, gases, or alcohol. Interestingly, 52% species (308/591) occurred in both rotten apples and grapes (Fig. 5c and Supplementary Dataset), suggestive of a highly overlapping composition of rotting fruit-associated bacterial community.

Based on the phylogenetic richness of species, we employed by reconstruction of unobserved states (PICRUSt) analysis to predict the functions of the decaying fruit-associated bacterial community using the Kyoto Encyclopedia of Genes and Genomes (KEGG). The top predicted functions were involved in carbohydrate metabolism, amino acid synthesis and DNA replication at the 1^st^ level, and in membrane transport, carbohydrate metabolism, replication, energy metabolism and translation at the 2^nd^ level (Fig. 5d,e). Most of the main functions were virtually associated with sugar metabolism, protein synthesis, and bacterial proliferation. Albeit of the much more differentiated bacterial individuals in two samples, the number of overlapping genes absolutely dominated the total gene in both decaying apple and grape samples (Fig. 5f). These results suggested that differentiated communities composed of rotting fruits converged towards similar functions: carbohydrate metabolism, amino acid synthesis and bacterial proliferation. The predicted functions of rotting fruit-associated bacteria were also in lines with the ones of fermented fly food (Fig. 4).

### Requirement of microbiota for *Drosophila* development

**Figure 6:**
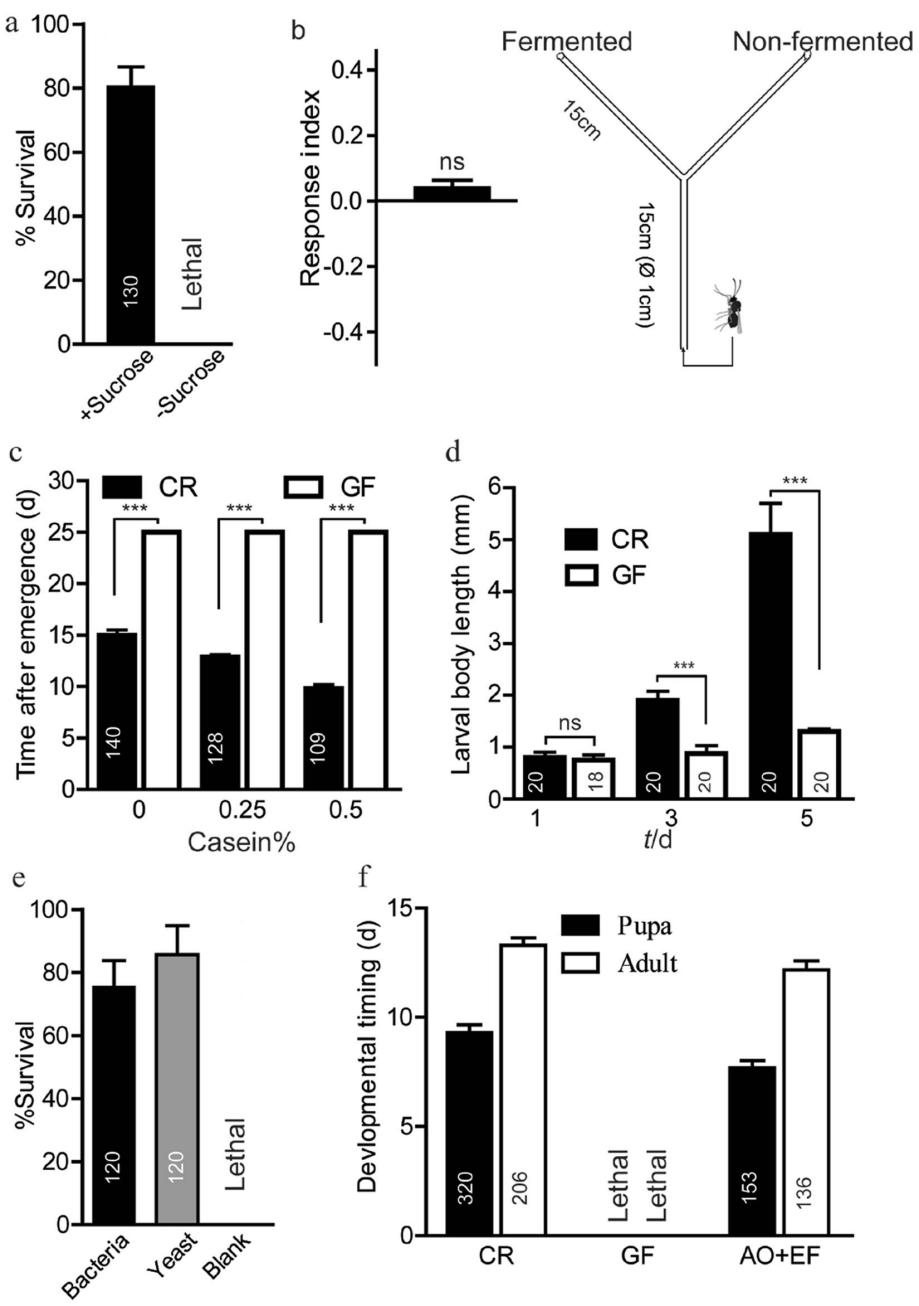
Commensal bacteria were essential for survival and fitness of *Drosophila*. (**a**) Sucrose was essential for *Drosophila* survival. 30 eggs were placed in the casein-cornmeal-agar media with (+Sucrose) or without sucrose (−Sucrose) in 6-mm Petri dishes. The eclosed adults were counted for survival ratio. (**b**) Fermentation was not required to confer protection against endoparasitoid wasps. Schematic drawing of the Y maze olfactory assay used for behavioral experiments with the wasp *Leptopilina boulardi*. Thirty wasps were placed at the bottom of the Y maze with a choice of fresh or fermented grape juice and wasp counts from each branch were made after 20 min. The response index of *L*. *boulardi* in the Y maze olfactory assay. (**c**) Microbiota facilitated the timing of adult emergence. Germ free (GF) eggs were transferred to autoclaved vials to generate GF flies, while GF eggs were replenished with mixed bacteria to conventionally reared (CR) flies. The timing of adult emergence was recorded in a cornmeal media containing casein over time (the cutoff for GF flies was arbitrarily assigned as 25-days). (**d**) Microbiota promoted the larval growth. The length of larval bodies was measured at day 1, 3, 5 ALE. (**e**) The source of microbes solely supported *Drosophila* survival. Thirty eggs were placed in the agar media with (Blank) or without microbes (Bacteria, Yeast), and the eclosed adults were counted for survival ratio. (**f**) The timing of pupa formation and adult emergence of flies in grape vials was recorded. CR flies developed from eggs without sterilization. GF eggs were transferred to vials with sterile grapes, while *Acetobacter* and *Enterococcus* (AO + EF) were replenished in vials. The timing of pupa formation and adult emergence was recorded, respectively. ANOVA tests with LSD post hoc analysis. Mean ± SEM. Symbols: NS p > 0.05; **p < 0.01; ***p < 0.001.

The egg-laying avoidance to the sucrose-rich sites conflicts with the universal rule that calorie-dense foods provide more essential nutritional value for animals. Indeed, embryos failed to survive fly food deprived of sucrose, while they normally developed in the presence of sucrose (Fig. 6a). Therefore, we predicted that this behavior was selected by virtue of predation avoidance in the wild. Given that the endosymbiotic bacteria, *Spiroplasma poulsonii*, offer protection against wasps^23,24^, we asked whether LAB could generate a natural barrier against them. To this end, we used a Y maze assay to examine the selection of *Leptopilina boulardi* confronted with fresh and fermented grape juice as described previously^23^. However, wasps did not show significant repellence to fermented grape purée, with a response index of 0.04 (Fig. 6b). Thus, it was unlikely that fermentation could protect flies against wasp parasitization, consistant with the natural observation that wasps efficiently infect with larvae inside rotten fruits.

Alternatively, the egg laying preference for fermentation was selected by nutrition balance. To address it, we interrogated whether the medium containing commensal bacteria could be more nutritive for developing *Drosophila* larvae than sterile ones. Resident microbes collaborate to digest complex substrates, synthesize nutrition, and even stimulate the excretion of digestive enzymes of hosts^25^. To understand the putative contributions of microbiota to systemic growth of hosts, we assessed the developmental timing of pupa formation and adult eclosion with laboratory and natural food, respectively. We artificially removed microbes on the surfaces of eggs and generated germ-free (GF) flies as described^18^. Conventionally reared (CR) flies normally developed in rich media with 0.5% and more casein (Fig. 6c). Conversely, none GF larvae developed into pre-pupal larvae or pupa even in rich media (Fig. 6c, the cutoff for the experiment was arbitrarily assigned as 25-days). This result indicated that larvae significantly relied on their commensal microbiota in casein-sucrose-cornmeal food, in agreement with published data^18^. In addition, microbiota were required to promote the size of larvae (Fig. 6d). Next, we asked whether flies could survive in the presence of microbiota, without cornmeal and casein. Surprisingly, fly eggs succeeded to form pupae and adults on agar plates with the addition of yeast and bacteria (Fig. 6e), indicating that microbiota were essential for the survival of developing larva. Attractively, GF flies did not survive sterilized grapes and never developed beyond the second instar stage (Fig. 6f), while CR siblings formed pupa and adults at 9.3 and 15.1 days old, respectively. Moreover, GF eggs replenished with the mixture of *Enterococcus* and *Acetobacter* formed pupa and adults at 7.7 and 14.6 days old, respectively (Fig. 6f). The results indicated that commensal microbiota were integral for the growth and development of *Drosophila*. However, the hatching rate of eggs on fermented halves did not significantly differ from their sterile counterparts (Supplementary Figure 3), suggesting that microbiota were not required for the development of embryogenesis. It could be partially explained by the fact that the inner yolk is the main source of nutrition for the embryo. In sum, *Drosophila* egg-laying preference for fermentation may reflect a postembryonic benefit in bacterial nutrition.

### Conservation of the oviposition preference for fermentation

**Figure 7:**
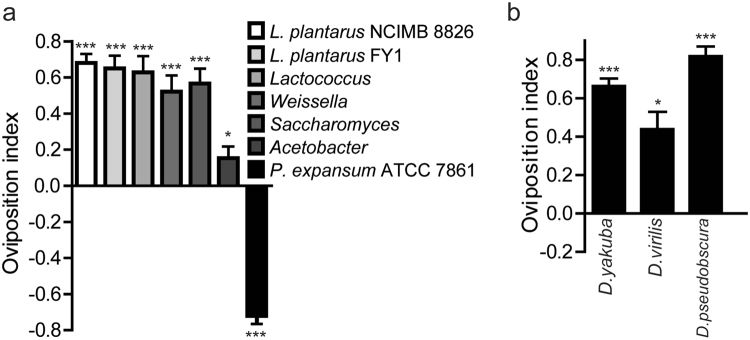
Conservation of egg-laying preference for commensal bacteria. (**a**) The oviposition index of *D*. *melanogaster* for a diet fermented by commensal and pathogenic microbes. The one-sample *t*-test was used to assess the mean deviance of each column from 0, n = 4–7. (**b**) The oviposition preference for EF fermentation was conserved in *drosophila* species. The one-sample *t*-test was used to assess the mean deviance of each column from 0, n = 5. Mean ± SEM; Symbols: NS p > 0.05; *p < 0.05; **p < 0.01; ***p < 0.001.

Natural *Drosophila* populations harbor many bacterial genera, including *Enterococcus*, *Lactobacillus*, and *Acetobacter*, and fungi *Saccharomyces* ^9,12^. Firstly, we sought to examine the general oviposition preference for fermentation with commensal microbial symbionts available. As with *E*. *faecium*, our data showed that *Drosophila* was robustly attracted to other LAB including *Lactococcus*, *Lactobacillus* and *Weissella*. In agreement with published data^26^, *Drosophila* was strongly attracted to yeasts *Saccharomyces*, and moderately attracted to acetic acid bacteria (Fig. 7a). These results suggested that the oviposition preference for fermentation might be a much more general theme in indigenous bacteria. However, *Drosophila* was robustly repelled by harmful mold *Penicillium expansum* (Fig. 7a), because pathogens produce toxicants, like geomycin, that impose risks on the fly. Combined with recent studies^26^, these results implied that *Drosophila* distinguished commensals from pathogens, and selected commensals-enriched sites for egg laying.

According to ecology, more than 3000 species of *Drosophila* and related genera inhabit all of the continents except Antarctica^25^. To shed light on the origin and evolution of this fermentation-induced behavior, we turned to a comparative approach of *drosophilid* species. We found that closely related *Drosophila* species have evolved a resembling preference to lay eggs on fermented food (Fig. 7b). Combined with recent studies that *Drosophila* deposit eggs mostly in the rotten fruits^27^, the results suggest that *Drosophila* has evolved a conserved strategy of oviposition in the wild.

## Discussion

*D*. *melanogaster*is intricately linked to environmental and symbiotic microbes in nature. In this study, we showed that microbiota, an ecological input, influenced the interesting behavioral outputs of *Drosophila* egg laying attraction. *Drosophila* chose the favorite egg laying sites by sensing sucrose with its receptors. To females, sucrose consumption by bacteria imparts ecologically relevant information regarding rich commensals that facilitate survival and fitness of offspring. To our best knowledge, our study is the first to identify the consequences of food taste changes from bacterial metabolism on animal egg laying behavior, gaining an insight into how commensal bacteria influence brain functions and behaviors.

All metazoans are associated with environmental and symbiotic microbes in a world^28^. In the wild, *Drosophila* feeds on overripe or rotten fruits that contain large amounts of fermenting microbes. Bacteria invade and colonize the soft tissues of overripe fruit, and the fate of rich sugar is degraded during fermentation (Fig. 4a), which eventually contributes to the high nutritional value of rotten fruit for saprophytic animals, like *Drosophila*. During ingestion, *Drosophila* acquires polymicrobial mixtures of bacteria in a great variety of habitats, and sustains their microbial gut community. In turn, *Drosophila* acts as a vector and promotes dispersal of the microbial cells in the environment, forming a coevolutionarysymbiosis in nature^29^. Thus, a large consortia of *Drosophila* microbes are represented by the bacterial genera *Lactobacillus*, *Acetobacter* and *Enterococcus* ^9,12^. Commensals are vertically transmitted to progenies via the deposition of contaminated mother’s faeces on the surface of the embryo and the surrounding substratum. It has been largely established that intestinal bacteria communities affect the two potent aspects of metabolism and immunity in hosts^30^. However, emerging innovations have sparked the notion that microbiota affect a wide array of brain functions ranging from neurodevelopment to its disorders, and social behavior^6,31^, proposing that indigenous microbes influence the central nervous system and behaviors under certain health states. Indeed, studies have shown that the commensal microbiota of mouse guts altered their neurological functions, leading to effects on mood and behavior^32^. In laboratory-reared *Drosophila*, it was found that indigenous bacteria, *Lactobacteria*, altered the mating preference of hosts^33^. How indigenous or commensal microbes influence *Drosophila* behaviors in the wild is being appreciated in our study. Our data revealed that commensal LAB act as potent modulators of oviposition selection, leading to a better understanding of the ecological relationships between the microbial and metazoan worlds.

Selecting a suitable site to deposit their eggs is an important reproductive requirement of *Drosophila* females, because eggs are vulnerable and larvae have limited motility. The hypothesis of ‘mother-knows-best’ stipulates that female oviposition decisions evolved to lay eggs in places with the best survival of offspring^34^. The egg laying behavior comprises three steps—an ovipositor motor program, a clean/rest period and a search-like behavior^2^. Oviposition decisions need multiple sensory modalities, such as visual, olfactory, gustatory and proprioception. The neural circuits by which bacteria mediate *Drosophila* oviposition preference are likely to be complex. It was postulated that *Drosophila* has an innate positional repulsion toward the odors of fermented diet (Fig. 2). However, it is conceivable that the oviposition attraction for a fermented diet overrode this positional repulsion if egg laying repulses occurred, consequently forcing females to leave the sterile sites. We found that the gustatory system was required for the decision-making process of ovipostion preference for fermentation (Fig. 2d,e). In higher-order brain regions, a group of projection neurons express an insulin-like neuropeptide integrated signal from the peripheral sensory systems and participate in the neural circuitry that underlies egg-laying site selection^2^.

Studies have shown that sucrose executed a crucial role in influencing the decision-making process of oviposition (Fig. 7a)^2^, but the ecological significance has yet to be acknowledged. It seems paradoxical that females would select energy-scarce sites to lay eggs, because energy essentially sustains the growth and development of any organism. It was proposed that the avoidance of egg laying on sucrose-rich media has been selected for by virtue of predation avoidance and larval dietary balance. Our data showed that fermentation is unable to protect flies against wasp parasitization (Fig. 6b), alternatively supporting the nutritional benefits of microbiota. Bacteria frequently dominate decaying fruit and consume sucrose/sugars (Figs 3 and 4), which could serve as an indicator of the amount of bacteria. Thus, fruit flies discriminate decaying fruit from fresh fruit by probing the concentration of sucrose, and finally locating favorable egg-laying sites associated with an abundance of bacteria. Albeit of less energy, fermenting bacteria are virtually required to facilitate the growth of *Drosophila*. Our data shows that indigenous bacteria stimulate the development of flies with both a laboratory and natural diet (Fig. 7). The selection of sites rich in bacteria could be due to the following aspects. First, bacteria collaborate to digest complex substrates and synthesize essential nutrients, such as bacterial proteins and vitamins that are essential for hosts. Second, bacteria stimulate the digestive systems of hosts. Commensal bacteria stimulate the execration of enzymes that facilitate nutrient digestion and absorbance in *Drosophila* intestines, assisting in intestinal homeostasis^35^. Therefore, our finding explains the conflicting observation that *Drosophila* selects sites that are energy-scarce, but have an abundance of bacteria to deposit their eggs. In nature, survival and fitness strategies should be made in the context of systemic ecology, in which flies and their indigenous bacteria collaborate to maximize the utility of finite resources. Our hypotheses is that the following process: (1) bacteria readily grow on overripe fruit, (2) bacteria metabolize sugar and reduce the concentration of sucrose in rotting fruit, (3) flies seek favorable sites for egg laying by activating sucrose receptors, (4) flies navigate to low-sugar sites that are linked with a rich source of bacteria, and lay eggs, (5) fermenting bacteria promote the development of larvae. Thus, our results reveal that sucrose acts as a cue that triggers egg-laying females to locate the bacteria-enriched sites.

Using the *Drosophila* model system, we revealed a natural ecological phenomenon whereby indigenous microbiota were required to regulate the egg-laying behavior of hosts. Molecular and genetic studies of *D*. *melanogaster* and microbiota could serve as a paradigm for other animal behaviors and microbiomes in nature. Future studies that evaluate the ecological mechanism underlying a range of behaviors and microbial communities would improve our understanding of the ecology of host-symbiont interactions.

## Materials and Methods

### Stocks and genetics

All fly stocks were cultured at 25 °C, 60% humidity in a 12/12 h light/dark cycle on standard cornmeal-yeast-sucrose food unless otherwise noted^36^. The *Oregon R* and *Canston S* strains were used as the wild-type strains. *Gr5a* [*ΔEP(X)−5*] and *Gr64a* ^2^ mutants were kindly gifted by Dr. Dahanuhkar (University of California, Riverside, UAS); *D*. *pseudoobscura* was gifted by Dr. Jian Lu (Peking University, China); *UAS-NaChBac* was gifted by Dr. Yufeng Pan (Southeast University, China). *Gr33a* ^1^, *Orco* ^2^, *IR76b* ^1^, *ninaB* ^1^, *UAS-Kir2*.*1*/+, *Gr5a-GAL4* and *Gr64a-GAL4* mutants were from the Bloomington *Drosophila* Stock Center for *Drosophila* strains; *D*. *yakula*, and *D*. *viliris* came from the Core Facility of *Drosophila* Resource and Technology, Shanghai Institute of Biochemistry and Cell Biology, CAS, China.

### Bacteria culture and counting

Commensal bacteria used in this study were listed and described in Supplementary Table 1. Bacteria were from China General Microbiological Culture Collection Center, and isolated from *Drosophila* using selective media^37^, and identified based on the 16 S rRNA sequence with the PCR primer set (F: 5′-AAAGATGGCATCATCATTCAAC-3′, R: 5′-TACCGTCATTATCTTCCCCAAA-3′). To culture commensal bacteria, selective media were used to assay the bacterial population of *L*. *plantarum* and acetic acid bacteria^38^. *E*. *faecalis* was cultured in 200 ml of liquid YCFA medium with 0.25% glucose^36^. In order to assay the bacterial population, agar YCFA medium with 0.25% glucose was used.

### Food fermentation

The media to assay behavior was a simple cornmeal-casein-agar food containing 1.5% casamino acids (Oxoid), 7.0% cornmeal, 5% sucrose and 1.5% agar^18^. For fruit assay, sucrose was replaced with 20% (v/v) grape and watermelon purée, respectively. Food media were autoclaved at 121 °C for 20 min, and then poured into the dishes. For food fermentation, the total 10^8^ CFU of bacteria were suspended in sterile 1x PBS and seeded onto fly food plates. Fly food plates were incubated at 36 °C for 36–48 h for fermentation.

### Oviposition preference assay

The 2-choice apparatus was assembled using a transparent 80-mm column with a 60-mm Petri dish at the bottom^17^. The 2-choice dishes were generated by evenly dividing food into two halves with a razor blade, and hand-puzzling 2 types of food in one dish by hand. For each test, 20 newly-eclosing females were collected and mated for two to three days with yeast paste. Flies were gently transferred into the assay cage without CO_2_ anesthesia, and allowed to lay eggs for 16 h in the dark. To assess oviposition preference, the amount of eggs on each half was counted, and an oviposition index (OI) was determined: [OI = (NO. of eggs laid on experimental food – NO. of eggs laid on control food)/total NO. of eggs laid]. For the oviposition assay with supernatants, fermented casein-cornmeal-sucrose medium were centrifuged at 12,000 rpm for 5 min, and supernatants were transferred and plated on the surface of fly medium and dried at 36 °C for 45 min. For the sucrose-rescue assay, fermented plates were frozen at −70 °C in a freezer overnight to terminate fermentation, and then warmed at room temperature for 2 h. Plates were added with 500 μl volumes of 0.4 M, 0.8 M, 1.2 M sucrose solution on the surface, and incubated at 36 °C for 45 min to vaporize. The 2-choice apparatus was assembled as above. For whole-force assays, groups of 2 female and 5 male flies were briefly transferred to cages, where they were allowed to lay eggs for 16 h. Flies were removed and laid eggs were counted. Egg laying was calculated by dividing the number of eggs by the number of living females at the end of the assay.

### Position preference assay

For positional preference, the number of flies on each half of the 2-choice dish was counted at 5-min intervals for 2 h as described^17^. For positional preference, the number of flies on each half of the dish was counted at 5-min intervals for 2 h with camera. The number of flies was totaled, averaged, and a position index (PI) was calculated: [PI = (NO. of flies on experimental food – NO. of flies on control food)/total NO. of flies on food]. For surgeries, females were anesthetized with CO_2_ on the pad, and antenna and forelegs were removed with fine forceps. Flies were allowed to recover for 2 d before testing.

### Feeding preference assay

To assay feeding preferences, the food mixing protocol was performed as previously described. In brief, Erioglaucine (FD&C Blue #1) was mixed into the experimental (Fermented) or control (H_2_O) food. Twenty mated females were allowed to feed for 4 h, after which they were frozen. Flies were then homogenized in 200 μl of PBS, and the homogenate was centrifuged at 8,000 g for 10 min. The absorbance values of the supernatant were measured at 625 nm and converted into the concentration of dye. Subsequently, feeding preferences was computed analogously to the oviposition preference index. For the capillary feeding assay, females were starved for 8 h, and then transferred to the vials with nylon plugs. Two capillary tubes supplying fresh or fermented grape juices were inserted through the plug. The volume of the juices consumed by flies was recorded. A feeding index (FI) was calculated: FI = (volume of fermented juice – volume of control juice)/total volume of juice.

### Sucrose concentration assay

A ground diet with sucrose was supplemented with bacteria and maintained at 36 °C. Samples were collected at 12 h intervals and frozen at −20 °C. The concentrations of sucrose were assayed with corresponding commercial kits (Nanjing Jiancheng Biotechnology Co. Ltd. Nanjing, China).

### Pyrosequencing and PICRUSt analysis

Samples of rotting apples and grapes were collected from orchards during the fruit harvest season, and sent to the Novogene Bioinformatics Technology Co., Ltd (Beijing, China). Total bacteria DNA extraction and sequencing was performed in accordance with standard protocols. Briefly, DNA was amplified using the 515 f/806r primer set (515 f: 5′-GTG CCA GCM GCC GCG GTA A-3′, 806r: 5′-XXX XXX GGA CTA CHV GGG TWT CTA AT-3′), which targets the V4 region of the bacterial 16 S rDNA. Pyrosequencing was conducted on an Illumina MiSeq. 2 × 250 platform according to published protocols^39^. Sample reads were assembled using mothur v1.32. Chimeric sequences were removed using the USEARCH software based on the UCHIME algorithm. The microbial diversity was analyzed using the QIIME software with Python scripts. Operational Taxonomic Units (OTUs) were picked using the *de novo* OTU picking protocol, with a 97% similarity threshold. Bacterial metagenome content was predicted from 16 S rRNA gene-based microbial compositions, and functional inferences were made from the Kyoto Encyclopedia of Gene and Genomes (KEGG) catalog, using the PICRUSt algorithm. The KEGG orthologies (KOs) were categorized into KEGG level 1 and 2 pathways.

### Survival, developmental timing and body length

For survival test of sucrose, 30 eggs within 10 h after egg laying were transferred to 60-mm Petri dishes casein-cornmeal-agar medium with or without sucrose. The adults were counted after 12 d, and the survival ratio was calculated. For the survival test of microbes, a similar process was carried out, except in a medium with suficient *A*. *orientalis* and *E*. *faecium* mixture or yeast. The process of making a germ free (GF) embryo was described with modification^18^. Briefly, we collected the eggs on the grape juice agar media within 10 h, and cleaned them with ddH_2_O to remove the yeast paste on the surface. Next, eggs were successively washed with 1:30 diluted sanitizer walch (Procter & Gamble Co., Cincinnati, OH, USA), and 2.5% hypochloride sodium (Sigma Aldrich, St. Louis, MO, USA), and 70% ETOH, and PBS containing 0.01% TritonX-100T. The absence of bacteria was verified by grinding eggs in sterile 1x PBS and spreading the suspension on LB, MRS, or Mannitol plates. Sterilized GF eggs were transferred to vials with autoclaved media within a biosafety cabinet. The GF fly system was supplemented with unknown or known bacteria to generate conventionally reared (CR) or gnotobiotic flies. For the grape assay, ripe grapes were incubated in 2.5% diluted hypochloride sodium for 30 min and then in 70% ETOH for 10 min to remove microbes on the surface. Sterilized grapes were pinched for the fruit test. Developing larvae were sampled at 1, 3 and 5 d post-oviposition, and were killed by placing them on a 65–70 °C heat block for 10–30 seconds until movement ceased. Images of heat-killed larvae were taken from the dorsal sides with a Leica DM4000 microscope. The body lengths were measured on ImageJ (http://imagej.nih.gov/ij/).

### Statistics

All statistical analyses were performed using SPSS 13.0 (SPSS Inc., Chicago, IL, USA). Specific statistical tests are noted for individual experiments. In behavioral experiments, a Shapiro-Wilk normality test determined whether the underlying data were consistent or inconsistent with a normal distribution. If consistent, a parametric test was used to evaluate differences; if inconsistent, a non-parametric test was used. Error bars in figures, mean ± standard error of the mean (S.E.M).

## Electronic supplementary material

Supplementary Information

bacterial taxa
