# Supplementary material for: Enterococci Mediate the Oviposition Preference of Drosophila melanogaster through Sucrose Catabolism
Source: Sci Rep. 2017 Oct 18;7:13420. doi: 10.1038/s41598-017-13705-5 (PMC5647369; doi:10.1038/s41598-017-13705-5)
Supplement: Supplementary file 1 — Supplementary Information [file 41598_2017_13705_MOESM1_ESM.pdf]

Enterococci Mediate the Oviposition Preference of *Drosophila melanogaster* through Sucrose Catabolism

Wei Liu [1,#,*], Ke Zhang [1,#], Yujuan Li [1], Wanzhen Su [1], Kunkun Hu [1,2], Shan Jin [2]

Supplementary

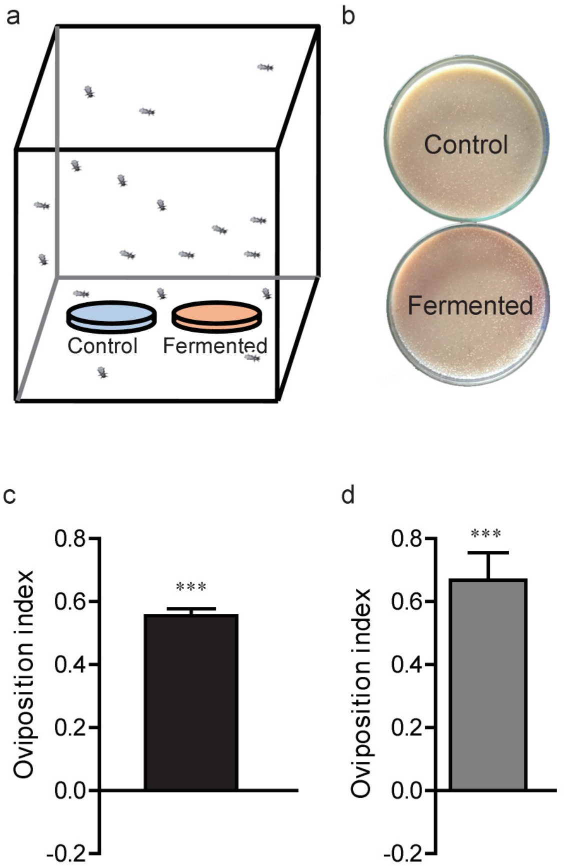

Supplementary Figure 1: The innate oviposition behavior in response to a fermented diet. (a) A diagram of the 2-choice cage with larger dimensions showing the sites available for egg laying. In the assay, 300 female flies were placed in cages (0.5 × 0.5 × 0.6 m) and given the choice to oviposit on two dishes of either control or fermented food. Oviposition dishes from each cage were replaced, and fly egg counts from each dish were made. (b) The representative result of egg laying in the 2-choice cage with larger dimensions. Top: control; bottom: fermented food. (c) The oviposition index in the

2-choice cage with larger dimensions. The oviposition index was calculated as (NO. of eggs laid on experimental food – NO. of eggs laid on control food) / total NO. of eggs laid. n = 7. (d) The quantification of egg laying preference for fermented media by virgin females. n = 6. The one-sample *t*-test; Error bars: SEM

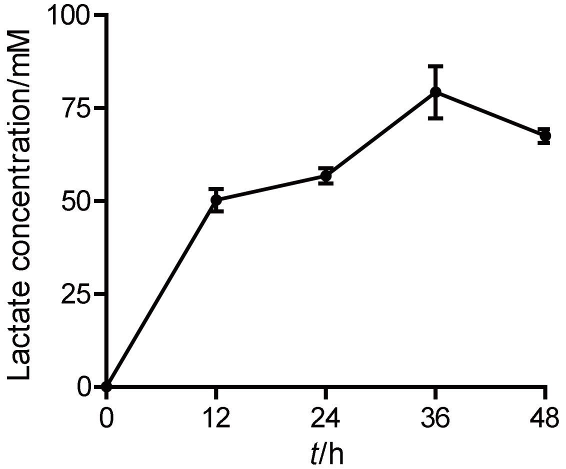

Supplementary Figure 2: The Dynamics of lactate production during fermentation. Lactate of fly food was detected using a commercial kit. Error bars: SEM

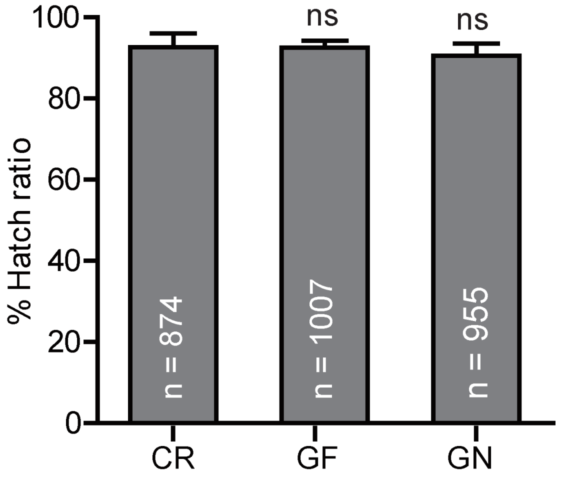

Supplementary Figure 3: The normal hatching ratio of GF eggs

The hatching rate of eggs was assessed after 24 h. The significance was calculated by ANOVA tests with LSD post hoc analysis and *P*-values were indicated. Error bars: SEM

Supplementary Table 1: Microorganisms and their sources used in this study

Organism (genbank no. availble)	Clade	Source
Lactobacillus plantarum NCIMB 8826	LAB	China General Microbiological Culture Collection Center, MRS culture collection
*Lactobacillus plantaru*s FY1 *KY038178*	LAB	*Wild-captured D. melanogaster* in Liu Laboratory, MRS culture collection
Enterococcus faecium KY990052	LAB	*Wild-captured D. melanogaster* in Liu Laboratory*,* YCFAG culture collection
Lactococcus lactis	LAB	*Wild-captured D. melanogaster* in Liu Laboratory*,* MRS culture collection
Weissella confusa	LAB	*Wild-captured D. melanogaster* in Liu Laboratory, MRS culture collection
Acetobacter orientalis FY1 KX943564	AAB	*D. melanogaster* in Liu Laboratory, NA culture
Acetobacter malorum	AAB	*D. melanogaster* in Handelsman Laboratory, NA culture
Saccharomyces cerevisiae	Yeast	YPD culture collection, gifted by Professor Pei Caixia in Shanxi Agricultural University, YPD cultrue
Penicillium expansum ATCC 7861	mold	China General Microbiological Culture Collection Center, YPD culture

LAB = lactic acid bacteria; AAB = acetic acid bacteria
